# A Mini-Two-Path Mach–Zehnder Interferometer Sensor with High Curvature Sensitivity Based on Four-Mode Fiber

**DOI:** 10.3390/mi16101149

**Published:** 2025-10-10

**Authors:** Wuming Wu, Jiayi Qian, Yuechun Shi, Xiaojun Zhu

**Affiliations:** 1 School of Engineering Science, Shandong Xiehe University, Jinan 250107, China; 2 School of Microelectronics and Integrated Circuits (Jiangsu Key Laboratory of Semiconductor Devices & IC Design, Package and Test), Nantong University, Nantong 226019, China; 3 Yongjiang Laboratory, Ningbo 315202, China

**Keywords:** mini-two-path, Mach–Zehnder interferometer, curvature sensitivity

## Abstract

We have proposed and presented a hybrid mini-two-path Mach–Zehnder interferometer (MTP-MZI) sensor based on four-mode fiber (FMF), where the reference path comprises of a section of a single-mode fiber (SMF), and the sensing path adopts a structure of SMF-FMF-SMF (SFS). Using arc discharge technology, the two paths are effectively fused and coupled, resulting in a robust MTP-MZI structure sensor. In the curvature detection, the maximum intensity sensitivity of curvature reaches 168.41 dB/m^−1^ when the curvature ranges change from 0 m^−1^ to 0.091 m^−1^. To the best of our knowledge, it is the highest curvature sensitivity in the MZI fiber sensor with intensity modulation. Furthermore, we also conducted a temperature-sensing experiment. The experiment results show that the maximum temperature sensitivity is only 78 pm/°C with a temperature range of 30–65 °C. The diverse exhibition of sensing performance for curvature and temperature enables us to effectively mitigate cross-sensitivity challenges. These results provide the experimental basis for developing a high-sensitivity sensor by employing the mini-two-path structure combined with specialty fibers.

## 1. Introduction

Curvature sensing plays a crucial role in mechanical engineering and structural health monitoring, including applications such as mechanical bending angle measurement [1], human posture detection [2], and bridge and road construction [3]. Compared to other electrical sensors [4], optical fiber curvature sensors offer several advantages, including small size, resistance to electromagnetic interference, corrosion resistance, high sensitivity, and low cost, making them widely used for curvature measurement. Generally, fiber curvature sensors can be categorized based on their modulation principles, such as intensity modulation [5], wavelength modulation [6], and frequency modulation [7]. In recent years, various fiber curvature sensors utilizing different optical devices have been proposed, including long-period gratings [8], fiber Bragg gratings (FBG) [9], MZI [10], and fiber Fabry-Perot interferometers [11]. Among these, MZI fiber curvature sensors have become a research focus due to their simple structure, compact size, high accuracy, minimal temperature crosstalk, and resilience in complex environments.

Traditional MZI sensors [12,13] function by dividing light into two separate paths using couplers. However, there has been a rapid transition from these couplers to inline MZI sensors. Various inline MZI curvature sensors have been proposed for different application fields, including curvature sensors for parametric monitoring [14] and large curvature sensors for health monitoring [15]. To enhance the sensitivity of fiber sensors, researchers have introduced different types of fibers, such as multimode fiber [16], coreless fiber [17], and multi-core fiber [18], which are widely used in the development of high-performance sensors. Recently, few-mode fiber (FMF) sensors have garnered significant interest owing to their distinctive advantages, such as structural simplicity, strong noise immunity, high sensitivity, and controllable mode properties [19]. These attributes make them promising candidates for various sensing applications, including refractive index [20], salinity [21], and displacement sensing [22]. However, challenges arise when few-mode fibers are directly coupled to SMFs due to mode mismatch, which hinders effective interference. In 2021, Guang et al. [23] demonstrated the use of the FMF for multimode interference and manipulation for beam shaping, highlighting its potential for advanced spatiotemporal manipulation. In inline MZI sensors, this issue can be addressed by modifying the sensor structure and utilizing designs such as spherical structures and core-offset structures. In 2018, Tong et al. [24] proposed an MZI sensor with melted balls at both ends of a few-mode fiber, achieving a temperature sensitivity of 0.054 nm/°C and a refractive index (RI) sensitivity of −27.77 nm/RIU. In 2021, Liu et al. [25] introduced an inline MZI sensor based on a few-mode fiber with a core-offset structure, which exhibited a salinity sensitivity of −0.27 nm/% and a temperature sensitivity of 0.15 nm/°C. To enable direct interference between few-mode fibers and SMFs, we combined the traditional MZI structure with inline MZI. In 2024, Zhu et al. [26] proposed an MTP-MZI for the first time, which realized interference directly through SMFs, achieving a curvature sensitivity of 112.6 dB/m^−1^ and a temperature sensitivity of 162 pm/°C. This innovative design significantly enhanced the interference effect and system adaptability by converting different beam modes through its parallel fiber paths configuration, thereby improving both interference effects and overall system performance.

In this paper, we propose an MTP-MZI sensor based on FMF. This sensor directly fuses an SFS and a section of SMF through discharge, forming a sensor that combines two paths with an inline MZI. The sensor is used for curvature and temperature measurements. It achieves a maximum curvature sensitivity of 168.41 dB/m^−1^ within the range of 0 m^−1^ to 0.091 m^−1^, which is the highest curvature sensitivity in the MZI fiber sensor utilizing intensity modulation, as far as we know. When the temperature changes from 30 °C to 65 °C, the maximum temperature sensitivity is 78 pm/°C. The different presentation of sensing performance between curvature and temperature avoids cross-sensitivity problems. The sensor boasts several advantages, including small size, robustness, high cost-effectiveness, easy packaging, and high sensitivity, providing an effective solution for multi-parameter and multi-dimensional MZI sensor detection. Additionally, it addresses the challenge of directly connecting few-mode fibers with SMFs for interference measurements.

## 2. Structure Design and Principle of the Sensor

**Figure 1:**
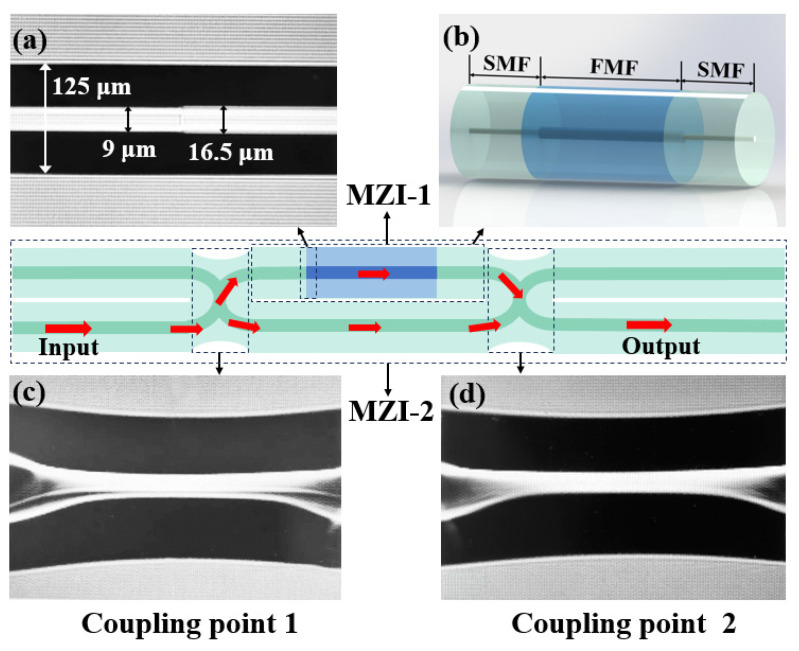
Schematic diagram of MTP-MZI. (**a**) Fusion diagram of SMF and FMF. (**b**) Structural diagram of MZI-1. (**c**) Coupling point 1 fusion diagram. (**d**) Coupling point 2 fusion diagram.

The proposed sensor can be manufactured using an optical fiber fusion splicer (Furukawa S178C, Tokyo, Japan), and the sensor schematic diagram is shown in Figure 1. The preparation process of the MTP-MZI based on FMF is as follows: First, a section of FMF is fused between two sections of SMF. The discharge mode of the fusion machine is adjusted to SM-MM mode, with a discharge setting of 120, and weld the SMF and FMF once through discharge, as illustrated in Figure 1a, which presents the fusion diagram of SMF and FMF. The cladding diameter of SMF is 125 μm, and the core diameter is 9 μm. The cladding diameter of FMF is 125 μm, and the core diameter is 16.5 μm. FMF is welded to another section of SMF to form the MZI-1. Figure 1b shows the structural schematic diagram of the MZI-1. Next, the MZI-1 and another section of SMF are placed side by side on the fiber retaining clip of the fusion machine. To achieve optical coupling between the two parallel SMFs to achieve the path structure, a stronger fusion process is required. The MM–MM mode with a discharge intensity of 250 is selected. All splicing parameters are finalized through systematic empirical optimization to ensure both stability and high reproducibility in sensor fabrication. A single discharge in manual mode can create a coupling point, as demonstrated in Figure 1c. Finally, the two optical fibers are moved a certain distance away from the previous coupling point, and the above steps are repeated to weld the second coupling point, as shown in Figure 1d. The arc discharge between the two optical fibers creates two coupling points, enabling the preparation of the MZI-2, which is MTP-MZI.

**Figure 2:**
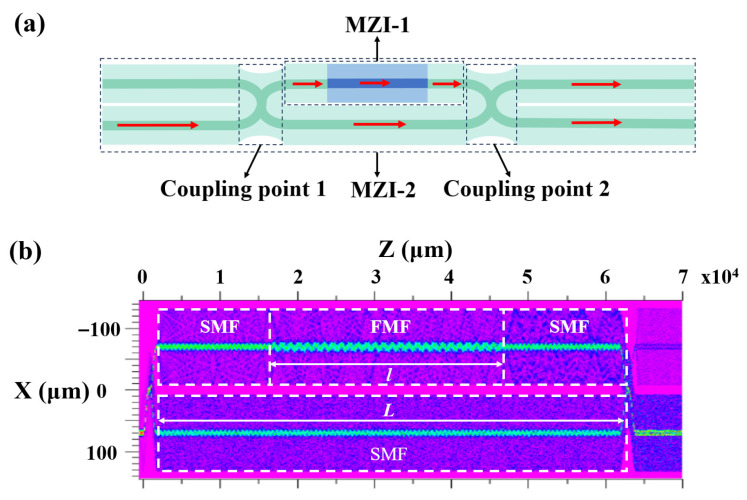
(**a**) Schematic diagram of optical path transmission of MTP-MZI. (**b**) Light field simulation diagram.

Figure 2a presents a schematic diagram of the optical path transmission in the MTP-MZI. When incident light travels through an SMF, it is split into two beams at coupling point 1. The path containing the MZI-1 structure is designated as the sensing path, while the other is the reference path. As the light enters the MZI-1 structure, it functions as an IMZI. Due to the mismatch in mode field diameters between the SMF and the FMF, the core fundamental mode light is excited, resulting in a part of light propagating in the cladding. These lights exist in both cladding and core as the cladding mode and core fundamental mode. When the FMF is fused with the SMF, the light traveling through the different paths generates interference. In the MZI-2 structure, the transmitted light is still predominantly governed by the core fundamental mode. When the light from the sensing path and the reference path converges at coupling point 2, interference occurs due to the differences in the transmission paths of the two beams.

To analyze the light distribution more effectively, we utilized the beam propagation method to simulate the MTP-MZI structure. The step-index FMF transmits the modes of LP01, LP11, LP21, and LP02, and with effective mode field areas of 190 μm^2^, 181 μm^2^, 201 μm^2^, and 182 μm^2^, respectively. The light field simulation diagram is shown in Figure 2b. In this figure, the MTP-MZI includes two coupling points used for beam splitting and coupling. The light splits at the splitting point and propagates along two distinct paths, and the light from both paths interferes at coupling point 2. As illustrated in Figure 2b, the sensing path exhibits oscillations in the light field distribution due to the fusion splicing of the FMF. This results in the excitation of high-order modes, which enables high-sensitivity environmental detection. The FMF length and the spacing between the coupling points are denoted as l and L, respectively. These parameters jointly determine the coupling ratio of light entering the two optical paths and the free spectral range of the interference spectra.

The intensity of interference light in the MZI-1 can be expressed as:

(1) $$I_{1}=I_{\mathrm{clad}}+I_{\mathrm{core}}+2\sqrt{I_{\mathrm{clad}}I_{\mathrm{core}}}\cos\varphi$$

where *I*_clad_ and *I*_core_ are the light intensity of the cladding mode and the surrounding core mode respectively. The phase difference between the cladding mode and the surrounding core mode is:

(2) $$\varphi =\frac{2\pi \Delta n_{1}l}{\lambda }$$

where $\Delta n_{1}$ is the effective refractive index difference between the cladding mode and the surrounding core mode. l is the interference length of MZI-1, and λ is the wavelength of the input light.

Based on the sensing principle of the MZI-1 above, the output light intensity of MTP-MZI can be expressed as:

(3) $$I=I_{1}+I_{2}+2\sqrt{I_{1}I_{2}}\cos\varphi$$

where *I*_1_ and *I*_2_ are the light intensities of the sensing path and the reference path respectively. The phase difference between the sensing path and the reference path is:

(4) $$\varphi =\frac{2\pi (n_{eff}^{1}-n_{eff}^{2})L}{\lambda }+\varphi_{0}=\frac{2\pi \Delta n_{eff}L}{\lambda }+\varphi_{0}$$

where $n_{eff}^{1}$ and $n_{eff}^{2}$ are the effective refractive indices of the sensing path and the reference path, respectively. Δ*n_eff_* is the effective refractive index difference between the two paths, and *φ*_0_ is the initial phase difference between the two paths. L is the interference length.

When the phase difference satisfies the condition *φ =* (*2i +* 1) *π*, *i* = 1, 2, 3, ···, the light intensity of the interference spectra reaches a minimum value, and the wavelength of the dry valley can be expressed as:

(5) $$\lambda_{i}=\frac{2\pi \Delta n_{eff}L}{2i+1}$$

where *λ_i_* is the wavelength of dry and valley. The wavelength separation between two adjacent interference valleys, called the free spectral range (FSR) of the MZI, can be expressed as:

(6) $$FSR=\lambda_{i-1}-\lambda_{i}\approx \frac{\lambda_{0}^{2}}{\Delta n_{eff}L}$$

where *λ*_0_ is the central wavelength.

**Figure 3:**
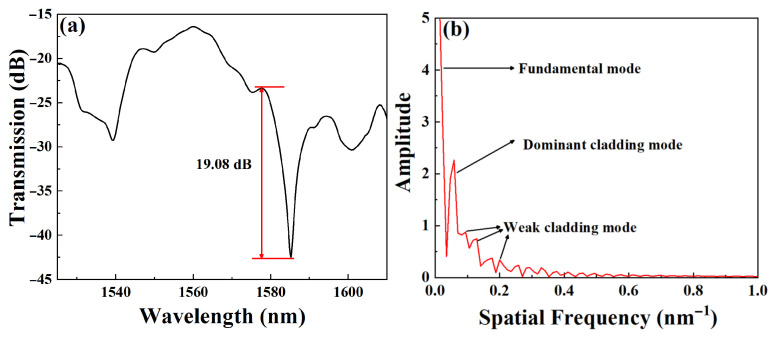
(**a**) Transmission spectra of the sensor and (**b**) spatial spectrum.

The two ends of the proposed sensor are connected to a broadband light source (BBS, OPEAK LSM-ASE-CF13, Tianjin, China) and a spectrum analyzer (OSA, Yokogawa AQ6370D, Tokyo, Japan) for real-time detection, respectively. Our experiments used an amplified spontaneous emission (ASE) source in the C + L band with an output spectrum of 1525 to 1610 nm. The resolution and sensitivity of the OSA used in the measurement were 0.02 nm and 80 dBm, respectively. When the l and L are divided into 1 cm and 2 cm, the transmission spectra of the sensor and the corresponding spatial spectrum distribution obtained by the Fast Fourier Transform (FFT) are shown in Figure 3a, which shows clear interference fringes with a high extinction ratio of 19.08 dB. It can be seen from Figure 3b that in the spatial spectrum, one dominant high-order mode and several weaker high-order modes coexist. The dominant high-order mode has a high energy percentage, which indicates that the MTP-MZI structure is very conducive to the excitation of high-order modes, giving the sensor higher detection sensitivity.

## 3. Experimental Results and Discussion

### 3.1. Curvature Measurement

**Figure 4:**
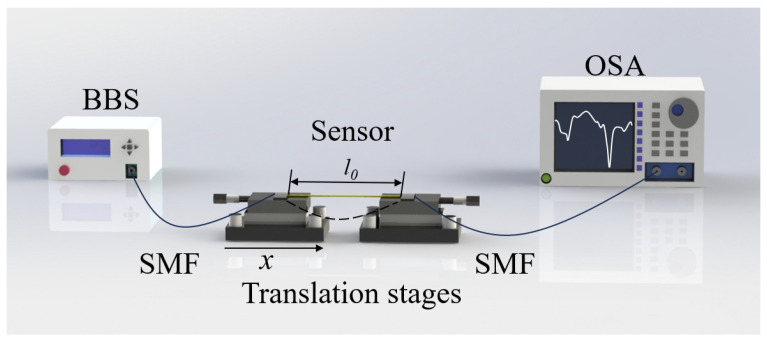
Schematic diagram of curvature testing device.

Figure 4 illustrates the experimental manufacturing schematic of the sensor for curvature detection. Both ends of the sensor are connected to BBS and OSA respectively for spectral observation. The sensor is fixed at both ends on two displacement platforms, maintaining a room temperature of 25 °C. By adjusting the distance between the left displacement platform and the right platform, the curvature of the sensor can be altered. It can be seen from the following equation:

(7) $$C=\frac{1}{R}=\sqrt{\frac{24x}{{\left(l_{0}-x\right)}^{3}}}$$

where *x* represents the displacement of the left displacement platform, *l_0_* represents the initial distance between the two displacement platforms, *R* represents the radius of curvature, and *C* represents the curvature. It should be noted that, to avoid the undesired effects of twist and stress on the curvature measurement, the optical fiber sensor is fixed onto a rigid fixture. The curvature is then applied to the sensor by controlling the curvature of this fixture.

**Figure 5:**
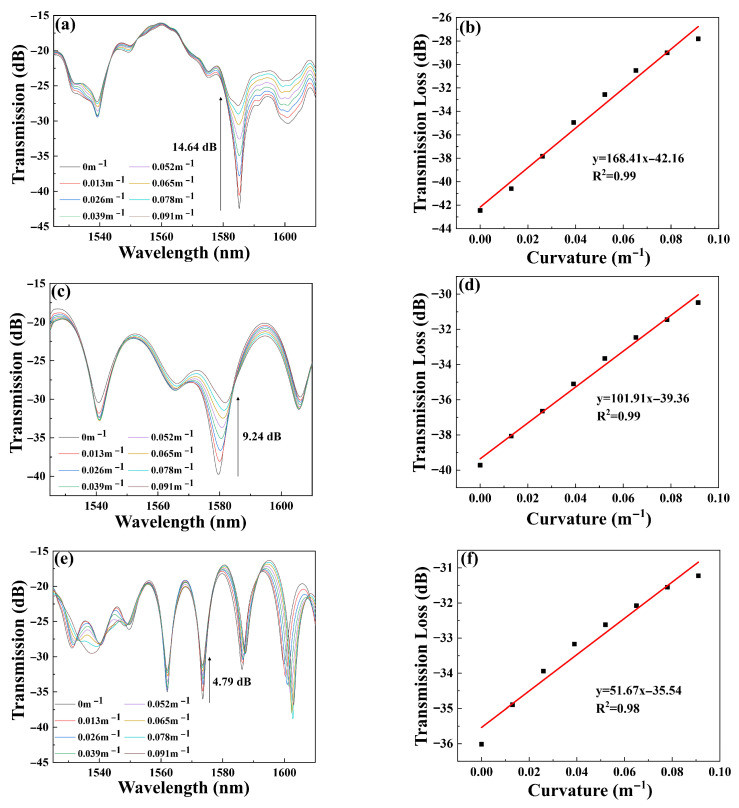
Curvature transmission spectra of (**a**) Sensor 1; (**c**) Sensor 2; (**e**) Sensor 3. Fitting curve of (**b**) Sensor 1; (**d**) Sensor 2, (**f**) Sensor 3.

To demonstrate the influence of the FMF length and coupling point spacing on the sensing characteristics, experiments were conducted on three sensors of varying lengths: Sensor 1 (l = 1 cm, L = 2 cm), Sensor 2 (l = 2 cm, L = 4 cm), and Sensor 3 (l = 3 cm, L = 6 cm). Figure 5 shows the transmission spectra and corresponding fitting curves for the sensors.

Figure 5a displays the transmission spectra of Sensor 1 at varying curvatures. Near 1580 nm, the intensity of the transmission spectra increases with changes in curvature. As the curvature gradually increases from 0 m^−1^ to 0.091 m^−1^, the intensity rises from −42.45 dB to −27.81 dB, resulting in a total increment of 14.64 dB. Furthermore, through the linear fitting of curvature and transmitted light intensity in Figure 5b, we conclude that the curvature sensitivity of Sensor 1 is 168.41 dB/m^−1^, with a linearity (R^2^) of 99.2%. This curvature sensitivity is currently the highest among the sensors tested, making Sensor 1 particularly valuable for curvature measurements and monitoring. Figure 5c presents the transmission spectra of Sensor 2. As the curvature changes from 0 m^−1^ to 0.091 m^−1^, the intensity alters from −39.72 dB to −30.48 dB, with a total increment of 9.24 dB. We can see from Figure 5d that the fitting curve for Sensor 2 indicates that the intensity curvature sensitivity is 101.91 dB/m^−1^, with an R^2^ of 99%. Figure 5e shows the transmission feature of Sensor 3 in the curvature detection. It is observed that when the curvature gradually increases from 0 m^−1^ to 0.091 m^−1^, the intensity rises from −36.01 dB to −31.22 dB, leading to a total increment of 4.79 dB. In Figure 5f, the curvature sensitivity and R^2^ for Sensor 3 are 51.67 dB/m^−1^ and 98%, respectively.

By analyzing the results in Figure 5, it can be concluded that Sensor 1 exhibits the highest curvature sensitivity, while the curvature sensitivity decreases as the sensor’s length increases. This trend can be attributed to the fact that light travels through two interference paths after passing through the beam-splitting point. As the interference path length increases, there is also a greater loss of high-order modes, resulting in reduced sensitivity. Therefore, it is essential to optimize the sensor length to balance the trade-off between sensitivity and interference path length, ensuring optimal performance for curvature sensing applications. It should be noted that the lengths of the FMF—1 cm, 2 cm, and 3 cm—were chosen to meet the requirements of device miniaturization and practical fabrication. By constraining the FMF length, the overall sensor dimensions could be maintained within a compact form factor. This selection also enabled a systematic investigation into how the FMF length influences sensor performance, while ensuring structural controllability throughout the experiments.

### 3.2. Temperature Measurement

**Figure 6:**
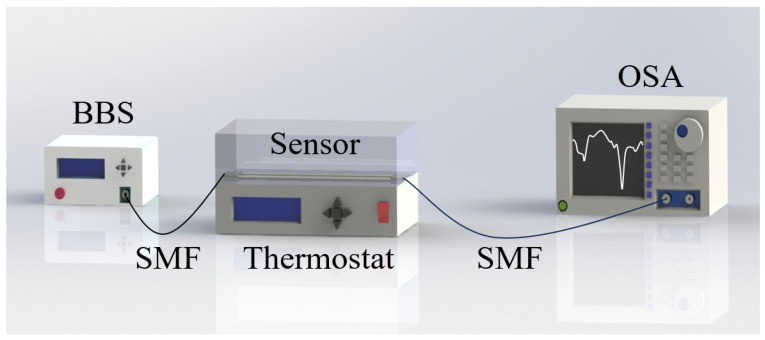
Schematic diagram of temperature testing device.

To ensure the reliability of the sensor in practical applications, it is essential to analyze its temperature characteristics. As part of our research, we conducted a thorough examination of the sensor’s temperature behavior. Figure 6 presents a schematic diagram of the temperature-sensing equipment used in our experiments. The sensor was placed in a thermostat, with one end connected to a BBS and the other end connected to an OSA. Notably, we maintained a stable curvature state of the sensor throughout the temperature detection process. Initially, we set the thermostat to 65 °C and gradually lowered the temperature to 30 °C. At each stable temperature point, we recorded the sensor data and allowed the system to stabilize for 10 min to ensure accurate temperature readings. Furthermore, we documented the data at intervals of 5 °C to gather comprehensive information regarding the sensor’s temperature characteristics.

**Figure 7:**
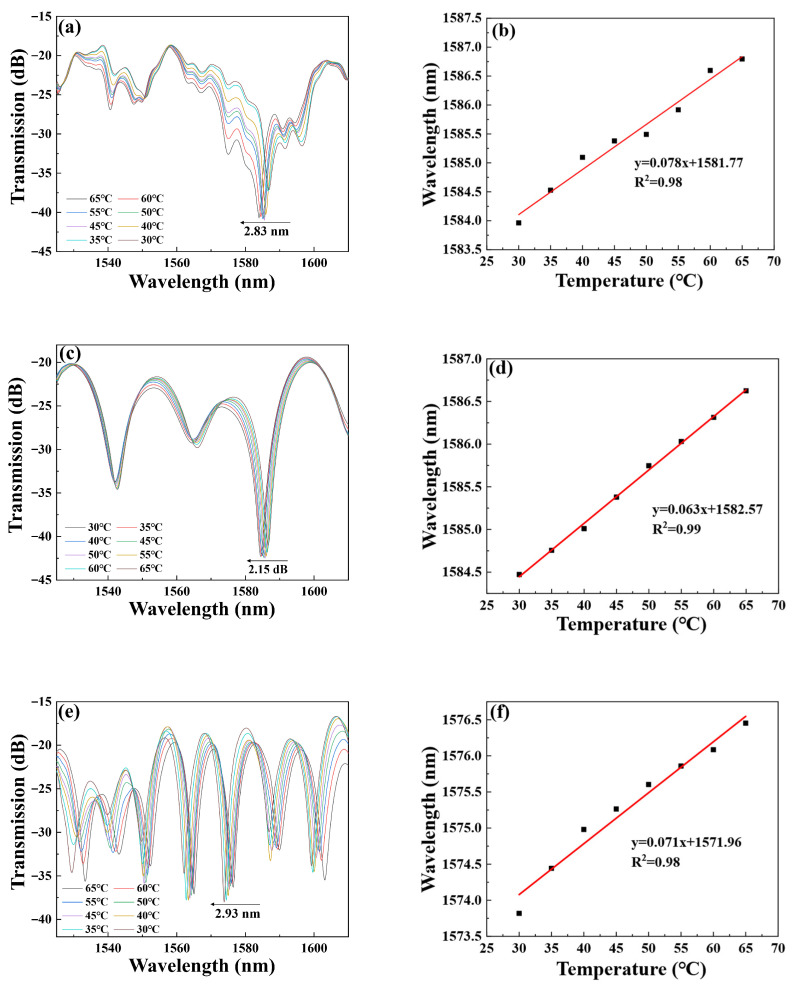
Temperature transmission spectra of (**a**) Sensor 1; (**c**) Sensor 2; (**e**) Sensor 3. Fitting curve of (**b**) Sensor 1; (**d**) Sensor 2, (**f**) Sensor 3.

Figure 7a illustrates the transmission feature of Sensor 1 when the temperature changes. The experimental results indicate that when the temperature drops from 65 °C to 30 °C, the wavelength has a blue shift from 1586.79 nm to 1583.96 nm, resulting in a total wavelength shift of 2.83 nm. To further quantify the temperature sensitivity of the sensor, we plotted a linear fit between wavelength and temperature, as shown in Figure 7b. Through the fitting analysis, we concluded that the temperature sensitivity of Sensor 1 is 78 pm/°C, with an R2 of 98%. Meanwhile, Figure 7c displays the transmission spectrum of Sensor 2 at various temperatures. When the temperature decreases from 65 °C to 30 °C, the transmission spectra have a wavelength blue-shift from 1586.62 nm to 1584.47 nm, resulting in a total wavelength offset of 2.15 nm. And the corresponding temperature sensitivity of Sensor 2 is 63 pm/°C, with an R2 of 99%, as shown in Figure 7d. Continue the temperature experiment on Sensor 3. When the temperature decreases from 65 °C to 30 °C, the wavelength of the transmission spectra blue-shifts from 1576.45 nm to 1573.52 nm, resulting in a total wavelength shift of 2.93 nm, as shown in Figure 7e. And the corresponding temperature sensitivity and R2 of Sensor 3 are 71 pm/°C and 98%, respectively, as shown in Figure 7f.

Through comparative sensing experiments on curvature and temperature, we observed that curvature variation primarily affects the intensity of the transmission spectrum, whereas temperature variation mainly causes a spectral shift. This behavior stems from the sensor’s two-path interference structure. Changes in curvature alter the power coupling ratio between the two paths, leading to a shift in the intensity of the interference pattern at specific wavelengths due to the modified balance of optical power between the interfering beams. In contrast, temperature changes induce variations in both the thermo-optic coefficient and the thermal expansion coefficient, ultimately resulting in a wavelength shift in the transmission spectrum [27].

By comparing curvature and temperature sensing, we found that the wavelength remained almost constant during changes in curvature. Thus, if only the intensity changes without any variation in wavelength, this can be interpreted as curvature sensing at a constant temperature. Conversely, if the wavelength does change, we can detect this shift to determine temperature fluctuations. Therefore, the cross-sensitivity between curvature and temperature can be managed by identifying losses in intensity and spectral characteristics. Specifically, in comparison to phase-shifted long-period fiber gratings (PS-LPFGs) [8], which address the cross-sensitivity between curvature and temperature by monitoring the separation between two wavelength dips. Our method offers a simple way to distinguish the two parameters. Furthermore, when compared to configurations using two fiber Bragg gratings (FBGs) that rely on their differential response to the same curvature [9], the proposed sensor achieves curvature detection in a more straightforward manner.

**Figure 8:**
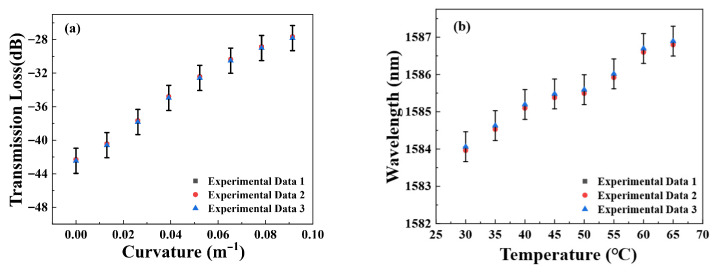
Repeatability tests of Sensor 1 under (**a**) curvature and (**b**) temperature variations.

To illustrate the stability and repeatability of the proposed sensor with curvature and temperature, we further analyze the spectral response of Sensor 1 when the curvature and temperature change back and forth three times, as shown in Figure 8. We can see that the transmission spectra only have a slight fluctuation when the curvature/temperature changes, which proves that the proposed sensor has excellent curvature/temperature stability, and can provide a practical application due to its reliable performance.

To emphasize the sensitivity of the proposed sensor, we compared the sensitivity results with those from previously reported studies with the intensity responses, as shown in Table 1. We found that the sensor’s curvature sensitivity significantly surpasses that of other previously reported sensors, making it the highest intensity curvature sensitivity currently available. The proposed sensor is capable of detecting small curvature changes with exceptional accuracy and stability, providing high-quality measurement data for practical applications.

## 4. Conclusions

In summary, this paper proposes and implements an MTP-MZI sensor based on FMF. The sensor demonstrates high sensitivity in curvature detection. The maximum curvature sensitivity was 168.41 dB/m^−1^ when the curvature range of 0 to 0.091 m^−1^. Additionally, when the temperature changes from 30 °C to 65 °C, the maximum temperature sensitivity reaches 78 pm/°C. The cross-sensitivity between curvature and temperature can be effectively managed by identifying intensity loss and wavelength shifts. The MTP-MZI structure sensors provide a novel approach to multi-parameter measurement and multi-dimensional detection thanks to their miniaturized structure. They can effectively address the challenge of no direct interference between few-mode fibers and SMFs, offering broad application prospects in fields such as knee joint detection and environmental monitoring.

## Figures and Tables

**Table 1:** Sensitivity comparison of sensors based on different structures.

Structure	Curvature Sensitivity (dB/m^−1^)	Extinction Ratio (dB)	Curvature Range	Temperature Sensitivity (pm/°C)	Reference
SMF-MMF-SMF	−130.37	−15	0.11–0.34 m^−1^	/	[16]
SMF (MTP-MZI)	112.6	1.856	0.005224–0.52245 m^−1^	162	[26]
SMF-MMF-Fiber sphere	−1.812	22.03	0–12.2894 m^−1^	78	[28]
SMF-DCF-SMF	15.19	10	0.98–1.753 m^−1^	79.8	[29]
SMF-MMF-Capillary-SMF	−9.25	~9	1.30–2.27 m^−1^	30	[30]
SMF-HCF-SMF	−4.28	~3	10.72–11.60 m^−1^	25.76	[31]
SMF-CLF-SCF-SMF	−26.5517	~23	0.527–1.395 m^−1^	75.3	[32]
SMF-Tapered MMF–SMF	−21.734	~26	0.7064–1.9129 m^−1^	6.1	[33]
**This study**	**168.41**	**19.08**	**0–0.091 m^−1^**	**78**	**This work**

## Data Availability

The original contributions presented in this study are included in the article. Further inquiries can be directed to the corresponding authors.
